# Personalization of Caffeine Therapy for Apnea of Prematurity: A Potential Role for Sensor Technologies?

**DOI:** 10.3390/s26123962

**Published:** 2026-06-22

**Authors:** Burcu Kolukisa Birgec, Beyza Toprak, Alexander Balfour Mullen

**Affiliations:** Strathclyde Institute of Pharmacy and Biomedical Sciences, University of Strathclyde, Glasgow G4 0RE, UK; burcu.kolukisa@strath.ac.uk (B.K.B.); beyza.toprak.2022@uni.strath.ac.uk (B.T.)

**Keywords:** apnea of prematurity, caffeine, respiratory, multi-modal sensor, precision medicine

## Abstract

Apnea of prematurity (AOP) remains a critical challenge in neonatal care, with caffeine citrate serving as the cornerstone of pharmacological intervention. However, the current standardized dosing schedule fails to account for significant inter-individual variability in caffeine pharmacokinetics and clinical response. This narrative review explores the transformative potential of integrating wearable sensor technologies and multi-modal data analytics into a closed-loop framework for personalized caffeine therapy. Based on a synthesis of current monitoring literature, we propose a theoretical, comprehensive monitoring system utilizing the area under the respiratory curve (rAUC) as a continuous proxy metric, alongside waveform amplitude analysis aligned with pediatric polysomnography standards. By incorporating emerging metrics such as respiratory rate variability (RRV) and hypoxic burden, the framework enables the objective quantification of respiratory stability. Furthermore, the integration of established neonatal intensive care unit (NICU) parameters for bradycardia and oxygen saturation detection provides a critical cross-validation layer to minimize artifact-induced false alarms. This conceptual model bridges the gap between advanced signal processing and clinical oversight, offering a scalable pathway toward precision dosing. By shifting from reactive to predictive neonatology, sensor-driven optimization can enhance therapeutic efficacy, reduce alarm fatigue, and ultimately improve developmental outcomes for preterm infants.

## 1. Introduction

Premature birth is defined as any birth that occurs before 37 weeks’ gestation. Globally in 2020, an estimated 13.4 million live births were preterm, representing approximately 1 in 10 of all births. It represents a considerable public health challenge and is the primary cause of death and disabilities in children under 5 years of age [1].

Preterm infants frequently stop breathing for short periods of time due to immaturity of the brain (central apnea), airway muscle weaknesses (obstructive apnea), or a combination of both (mixed apnea). This apnea of prematurity (AOP) results in recurrent episodes of intermittent hypoxemia (IH) that can potentially lead to complications such as bronchopulmonary dysplasia, retinopathy of prematurity, or neurodevelopmental delays [2]. Apnea of prematurity affects almost all premature infants born before 28 weeks, 50% of infants born at 30 weeks, and around 20% of infants at 34 weeks’ gestation but tend to resolve as the infant matures. Apnea of prematurity does not have a precise medical definition, but major authorities generally agree that a respiratory pause lasting ≥ 20 s or shorter pauses with concurrent bradycardia (<80–100 beats per minute) or oxygen desaturation (≤80–85%) constitutes an apneic event [3,4,5]. However, there is less consensus surrounding the potential cumulative impact of even shorter breathing pauses and their downstream impact on clinical outcomes in premature infants [6].

The mainstay of AOP treatment is to improve respiratory stability, prevent hypoxia and avoid mechanical ventilation which is associated with several negative outcomes such as delays in neurodevelopment, cardiopulmonary problems and respiratory failure [7,8,9]. To this effect, preterm infants with AOP are often provided with non-invasive respiratory support such as continuous positive airway pressure (CPAP) and pharmacological treatment with methylxanthines [10,11].

**Figure 1:**
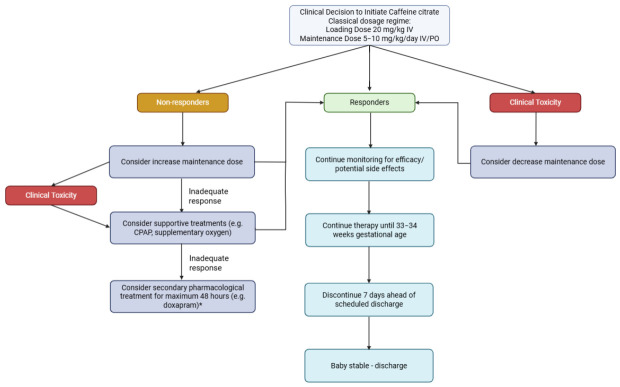
Treatment schema of Apnea of Prematurity. IV: intravenous; PO: per oral; CPAP: continuous positive airway pressure. * Doxapram is an unlicensed medication for apnea of prematurity. Note: This treatment flowchart represents a generalized clinical pathway. Specific interventions and thresholds may vary depending on national protocols and local institutional practices. A detailed summary of major guideline recommendations is provided in Section 2.

Methylxanthines, such as aminophylline, caffeine, or theophylline have been used for the treatment of AOP for over 50 years [12,13]. Caffeine citrate has become the preferred first line methylxanthine for treatment of AOP because of its wider therapeutic index and longer biological half-life that permits once daily administration (Figure 1). Caffeine acts as a selective adenosine antagonist at the A_2a_ receptors and a non-selective adenosine antagonist at A_1_ receptor. Post-administration of caffeine, positive pharmacological impacts are observed in the brain, lungs, and cardiovascular systems of preterm infants which result in improved respiratory drive, improved diaphragmatic contractility and function, reduced pulmonary inflammation, and positive hemodynamics [12,14,15,16,17]. The collective pharmacological effects of caffeine terminate or significantly reduce the incidence of apnea and initiate normal breathing patterns. Furthermore, caffeine administration increases the likelihood of CPAP being successful, supports earlier discontinuation from mechanical ventilation and reduces ventilator-induced lung injury [14].

The use of caffeine as the predominant treatment of AOP was consolidated following the Caffeine Therapy for Apnea of Prematurity (CAP) trial. This pivotal trial was the first randomized, placebo-controlled, multicenter trial of caffeine to study the short- and long-term efficacy and safety of caffeine therapy in infants with very low birth weight. Infants receiving an intravenous loading dose of 20 mg/kg caffeine citrate followed by a daily maintenance dose of 5–10 mg/kg had a lower incidence of bronchopulmonary dysplasia compared with placebo [11]. However, following on from the CAP trial, many questions on the optimal use of caffeine remain elusive, including treatment initiation, therapy duration, discontinuation, and appropriate drug monitoring methodology [14,18,19,20,21,22,23]. Interpretation of individual caffeine studies for the treatment of AOP has been hindered by the heterogenicity of infant populations and primary study outcomes. Furthermore, the pharmacokinetic profile of caffeine in preterm infants is complex.

This review seeks to address two primary questions: (1) What are the fundamental limitations of current neonatal monitoring systems and clinical parameters in accurately capturing continuous respiratory dynamics during AOP and its treatment with caffeine? and (2) how can the integration of wearable sensor technologies and emergent metrics, such as the area under the respiratory curve (rAUC), be leveraged to optimize and personalize caffeine treatment?

The primary contribution of this study is the proposal of a novel, theoretical proxy monitoring framework. By synthesizing the current literature on neonatal monitoring and caffeine pharmacodynamics, this study provides a conceptual shift in AOP management from traditional event-based counting towards a proactive, sensor-driven precision medicine approach.

## 2. Pharmacokinetics of Caffeine in the Preterm Infant

The standard dosing regimen from the CAP trial [11], 20 mg/kg intravenous caffeine citrate loading dose followed by a maintenance of 5 mg/kg caffeine citrate once a day either by intravenous or oral administration typically results in a therapeutic caffeine level of 5–20 mg/L [24]. Despite the universal acceptance of caffeine citrate, specific treatment criteria including patient eligibility, dosing regimens, therapeutic drug monitoring (TDM), and treatment duration, vary significantly among major health authorities. The European Consensus recommends caffeine citrate initiation for infants weighing < 1251 g [25], whereas the United Kingdom National Institute for Health and Care Excellence (NICE) [26] and the World Health Organization (WHO) [27] target infants born at ≤30 weeks and <37 weeks of gestation, respectively. Regarding pharmacotherapy, a standard loading dose of 20 mg/kg is widely accepted, with the notable exception of Queensland Health, Australia, which permits a broader range of 20–80 mg/kg [28]. Maintenance dose recommendations exhibit similar heterogeneity, ranging from a strict 5 mg/kg/day (WHO) [27] and 10 mg/kg/day (Australasian Neonatal Medicines Formular (ANMF)) [29], to flexible ranges of 5–10 mg/kg/day (NICE and European Consensus) [25,26] or 5–20 mg/kg/day (American Academy of Pediatrics (AAP) and Queensland Health) [5,28]. Notably, routine TDM is not universally advised, and only the AAP and ANMF recommend TDM, specifically when maintenance doses exceed 20 mg/kg/day or when clinical toxicity (blood concentrations > 60 mg/L) is suspected [5,29]. Finally, guidelines for treatment cessation also diverge with the AAP recommending discontinuation between 33 and 35 weeks postmenstrual age if the infant is clinically stable [5], whilst NICE requires an apnea/bradycardia-free period of 5–7 days or alternatively when reaching 33–34 weeks postmenstrual age [26]; and the WHO suggests cessation after 6 weeks of treatment, reaching >34 weeks postmenstrual age, or demonstrating no apneic events for more than 5 days [27].

The pharmacokinetic profile of the U.S. Food and Drug Administration (FDA)-approved caffeine medicine is summarized in Table 1 [30,31].

However, the pharmacokinetic profile of caffeine in infants is complex for numerous reasons. Preterm infants exhibit nonlinear development, leading to rapid maturational changes in body composition, liver, and kidney function, that have an impact on caffeine pharmacokinetics [32,33,34]. Furthermore, a recent review by He, et al. [35] has summarized other important covariates including birth weight, genetic polymorphism of A_1_ and A_2a_ adenosine receptors, concurrent medications, feeding patterns, and pathological conditions that additionally impact upon caffeine pharmacokinetic parameters. Maternal prenatal caffeine consumption can also impact on fetal adenosine receptor function since caffeine, and metabolites such as theophylline, cross the placenta and can accumulate in the fetal brain [36]. Consequently, babies born to mothers with high caffeine consumption may have an inherent pharmacological tolerance towards caffeine necessitating larger doses. All of the above pharmacokinetic and pharmacodynamic variables further emphasize the need for preterm infants who lack appropriate clinical response to standard-dose caffeine therapy regimes to require individualized dose optimization [2,33]. This seems particularly relevant for very low-weight preterm infants where pharmacokinetic modeling would suggest that these infants require higher-than-standard caffeine dosing to maintain caffeine plasma levels above the 15–20 mg/L concentration range considered optimal for treatment of AOP [37,38]. However, higher caffeine dosing regimens require more careful monitoring considerations to address the potential for toxic side effects such as seizures, arrhythmias, rhabdomyolysis, tachycardia, tachypnea, hyperglycemia, agitations, and metabolic acidosis [39,40,41]. If potential toxicity or persistent apnea is observed, then caffeine blood concentrations should be measured [40,42]. Routine therapeutic drug monitoring is however not recommended due to the risk of causing anemia in preterm infants [29,43,44]. Therefore, outcomes directed by physiological responses to caffeine therapy may afford better opportunities to support AOP.

## 3. Monitoring of Apnea of Prematurity

Continuous monitoring of heart rate (HR), respiratory rate (RR), and oxygen saturation (SpO_2_), are central in the provision of care for AOP [20]. Nevertheless, several limitations are noted with existing devices which constrain clinical practice [45,46].

Electrocardiography (ECG) is a non-invasive method widely applied in the neonatal intensive care unit (NICU) to identify bradycardic events associated with AOP [47,48]. In neonates, the use of ECG has several practical limitations, including skin irritation from adhesive electrodes, restricted infant movement due to cables, delayed signal response, and a high rate of false alarms [45,46].

Pulse oximetry is another commonly used non-invasive monitoring modality in NICUs, providing continuous estimates of peripheral oxygen saturation (SpO_2_) [49]. This technique uses photoplethysmography (PPG), to differentiate between oxyhemoglobin and deoxyhemoglobin [50,51,52]. Although widely implemented across clinical settings, pulse oximetry is subject to multiple sources of inaccuracy, including motion artifacts, skin pigmentation, perfusion status, sensor placement, and environmental light exposure [47,53,54]. In addition, its calibration is based on healthy adult populations, which may limit reliability in preterm infants. While pulse oximetry is typically reported to have an accuracy of ±2% (standard deviation), this corresponds to a potential error margin of up to ±4% within 95% confidence intervals. Weak correlations between peripheral oxygen saturation (SpO_2_) and arterial oxygen saturation (SaO_2_) during spontaneous fluctuations further question its reliability in dynamic neonatal conditions [50].

Thoracic impedance monitoring estimates respiratory activity by measuring changes in electrical resistance across the chest wall. While it is widely used in neonatal care and can detect central apnea, it reflects chest wall movement rather than true airflow, limiting its ability to accurately identify obstructive or mixed apnea and abnormal breathing patterns [45,46,48].

In routine clinical practice, apnea detection is, therefore, not solely device driven. When an alarm is triggered, healthcare professionals clinically assess the infant and document events, which are then used to subsequently guide clinical therapy. However, this is a labor-intensive process which is prone to many false positives. One study reported approximately 200 apnea events recorded by nurses and 415 events detected by bedside monitors, yet only around 8% and 50% of these events, respectively, represented true apneic episodes [55].

Furthermore, the susceptibility of conventional monitoring systems to motion artifacts contributes to a high frequency of false-positive alarms. This can result in alarm fatigue among NICU staff, reducing responsiveness to alerts and increasing the risk of missing clinically significant events. Reported nurse response rates to alarms in intensive care settings range between 33 and 46.9% [56,57,58].

A wide range of monitoring techniques using impedance-based systems, piezoelectric sensors, or nasal pressure devices have been employed in AOP research (Table 2). However, the absence of a consistent reference standard across studies has led to substantial variability in reported performance with wide sensitivity estimates ranges from 9% to 100%, reflecting differences in methodology and validation approaches [46,48]. Emerging non-contact methods have also been evaluated to detect respiratory and heart dynamics. Different sensors employing radar-based, sound-based, video-based, thermal imaging, or far-infrared imaging have been evaluated; however, the clinical translation of these contactless modalities is currently limited by high equipment costs and a lack of rigorous, structured validation in real-world clinical environments as opposed to the idealized validation environments where initial research is performed [59,60,61,62,63].

Integrating artificial intelligence (AI) software within medical device hardware shows promise to improve apnea identification and clinical decision-making. Currently employment of AI is limited by sensitivity to motion artifacts, restricted clinical validation in neonates, and challenges related to interpretability. In addition, the ‘black-box’ nature of many algorithms and limited access to raw physiological waveforms restrict both prospective and retrospective analysis, reducing transparency and clinical trust [64,65].

These limitations collectively highlight a fundamental gap in current neonatal monitoring. Existing systems are not designed to provide continuous, high-fidelity, and interpretable physiological data required to accurately assess treatment response. As a result, standardized caffeine dosing strategies remain poorly aligned with individual physiological variability. Consequently, there is a clear need for continuous, real-time, and individualized monitoring approaches capable of capturing respiratory dynamics with sufficient resolution to inform treatment optimization.

## 4. Continuous Proxy Monitoring: A Conceptual Framework

### 4.1. Proxy Monitoring and Metrics

In conventional clinical practice, the diagnostic criteria and monitoring thresholds for AOP remain fragmented and lack universal consensus among medical authorities. For instance, the defining duration of a pathological apneic pause varies significantly across guidelines, characterized as a cessation of breathing lasting anywhere from 10 to 20 s, ≥20 s, or simply the absence of two consecutive breaths. Similarly, bradycardia thresholds are inconsistently established, ranging from a heart rate drop below 80–100 beats per minute (BPM) to more stringent criteria such as <50 BPM for 5 s or <60 BPM for 15 s. Oxygen desaturation criteria are equally disparate while the American Academy of Sleep Medicine (AASM) defines a clinically significant event as a relative ≥3% drop from baseline, other neonatal authorities rely on absolute thresholds (e.g., SpO_2_ <80–85%) or subjective clinical observations like cyanosis and pallor [3,4,5].

Furthermore, there remains no universal consensus on severity grading for AOP, particularly regarding the frequency of respiratory events, bradycardia, and oxygen desaturation in preterm infants. Proposed threshold values vary considerably across studies and clinical guidelines. For instance, Rosen, et al. [66] defined a critical threshold as more than five significant events requiring intervention, whereas guidelines, such as those from the British Thoracic Society [67] are primarily designed for older pediatric populations (e.g., Obstructive Sleep Apnea <16 years old and Central Sleep Apnea in children aged 2–15 years), leaving a distinct diagnostic gap for preterm infants.

Crucially, existing guidelines along with most current wearable and AI-assisted neonatal systems do not incorporate continuous, comprehensive metrics, or robust severity scales, instead relying predominantly on discrete, binary event counting. By excluding hypopnea and short-duration respiratory pauses, this threshold-based approach fails to capture the true cumulative burden of respiratory instability and consequently, the extent of clinical deterioration in the infant.

Rub, et al. [6] highlighted the ambiguity surrounding definitions of apnea and hypopnea in AOP, raising a critical question: “Is a 15-s pause with heart rate dropping to 90 beats per minute clinically equivalent to a 10-s pause with heart rate to 70? Does the oxygen desaturation threshold of 80% versus 85% matter for long-term outcomes?”. Consequently, this profound lack of standardization raises an important question: are current fragmented metrics sufficient to guide critical clinical decisions, such as the initiation, titration, or discontinuation of caffeine therapy?

This profound lack of standardization suggests that fragmented metrics are insufficient to guide critical decisions regarding caffeine therapy. Current practice relies on static, weight-based dosing that fails to adequately capture inter-individual pharmacokinetic variability [2,32,68]. Therefore, next-generation monitoring systems must move beyond opaque, alarm-based frameworks. Emerging wearable technologies, such as the PneumoWave transparent system [69], demonstrate the feasibility of continuous, high-fidelity waveform acquisition.

Building on this concept, we propose a theoretical concept of ‘proxy monitoring’ as a clinical strategy in neonatal care. Proxy monitoring utilizes wearable biosensors to continuously assess respiratory dynamics, providing a non-invasive alternative to traditional apnea monitoring and therapeutic drug monitoring. Rather than relying solely on discrete event frequency, this approach evaluates the overall burden and stability of respiratory function. Changes in respiratory patterns could potentially serve as a pharmacodynamic proxy for monitoring the response to caffeine during treatment of AOP and able determine severity of AOP.

**Figure 2:**
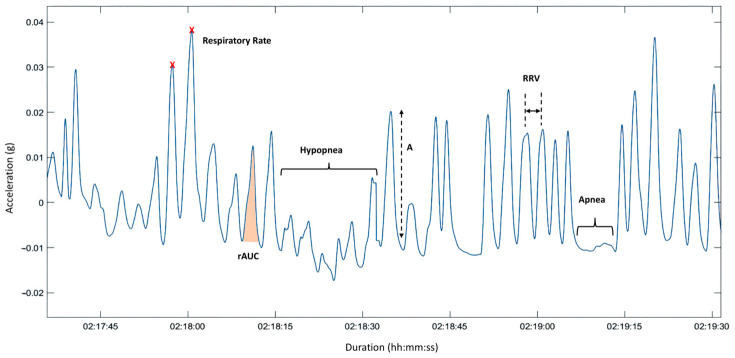
A theoretical example of respiratory signal captured using the PneumoWave device on a pediatric patient. rAUC: area under the respiratory curve, RRV: respiratory rate variability, t: time, A: amplitude. Red crosses show the count of peaks.

In this new theoretical paradigm, the area under the respiratory curve (rAUC) becomes a cornerstone of proxy monitoring concept and a potential ‘digital biomarker’ (Figure 2). The proposed rAUC quantifies the continuous dynamic spectrum of breathing effort. Preterm infants naturally exhibit irregular breathing with fluctuating waveform amplitudes. Following caffeine administration, respiratory instability decreases, which subsequently causes a reduction in waveform amplitude. While this reduction can be misinterpreted as declining respiratory drive, rAUC integrates both amplitude and time, accurately capturing the total respiratory effort. This allows quantification of clinical severity as it incorporates frequency, duration, and depth of events. The core novelty of the proposed rAUC proxy framework lies in its ability to quantify continuous physiological burden, offering more comprehensive and objective metrics rather than traditional count-based algorithms.

Analogous to clinical spirometry, where the area under the expiratory flow-volume curve assesses lung capacity [70,71], calculating the AUC of a wearable respiratory signal provides a dynamic estimate of respiratory efforts. This continuous rAUC analysis enables precise tracking of breathing effort and the early detection of hypopnea. Furthermore, aggregating rAUC with other high-resolution metrics such as Respiratory Rate Variability (RRV), amplitude changes, and the frequency, duration, and accumulation of apnea/hypopnea and period breathing creates a comprehensive ‘respiratory burden profile’ (Table 3). This composite profile directly reflects the activity of the brainstem’s respiratory control centers [72,73,74], the primary targets of methylxanthine therapy thereby providing a continuous physiological baseline for dynamic and individualized dose titration.

The feasibility of using continuous respiratory dynamics has been established in other clinical services. However, application of these metrics remains fragmented and rarely evaluated as an integrated whole in neonatology. For example, breathing rate is a core component of the Apgar score in pediatrics and the National Pediatric Early Warning Score (PEWS) for detecting clinical deterioration [75,76]. Similarly, monitoring of respiratory volume and patterns is already utilized to predict exacerbations in chronic adult conditions like COPD [77,78,79], demonstrating that subtle shifts in breathing dynamics often precede overt clinical collapse. Furthermore, it was found that RRV can be a good signal to detect deterioration on ICU [80]. Advanced respiratory biosensors are also validated for detecting opioid-induced respiratory depression [81,82]. If wearable technology can capture respiratory depression and exacerbations, it can similarly quantify the respiratory stimulation induced by methylxanthines. Similarly, the concept of hypoxia burden is widely utilized in obstructive sleep apnea, employing an area-under-the-curve approach to accurately characterize both the depth and duration of desaturation events [83,84].

Moreover, recent studies evaluating doxapram, an unlicensed second-line medication for AOP in preterm infants by using continuous data to objectively assess treatment response. Poppe et al. [85] used SpO_2_/FiO_2_ ratio and area under the SpO_2_ curve to detect treatment success and failure. Borenstein-Levin et al. [86,87] utilized a SpO_2_ histogram classification system to assess doxapram efficacy and respiratory support system by bedside monitoring. In a related short communication, Sur and Paria [88] outline this methodology and highlighted the related studies. These studies affirm that detailed data analysis can successfully evaluate treatment outcomes in AOP and could be equally utilized for caffeine therapy. Applying comprehensive, multi-modal metrics to caffeine treatment, the primary pharmacological intervention for AOP, is a necessary step. Adapting this framework for caffeine bridges the gap between drug administration and objective physiological evaluation.

### 4.2. Proxy Monitoring Algorithm

**Figure 3:**
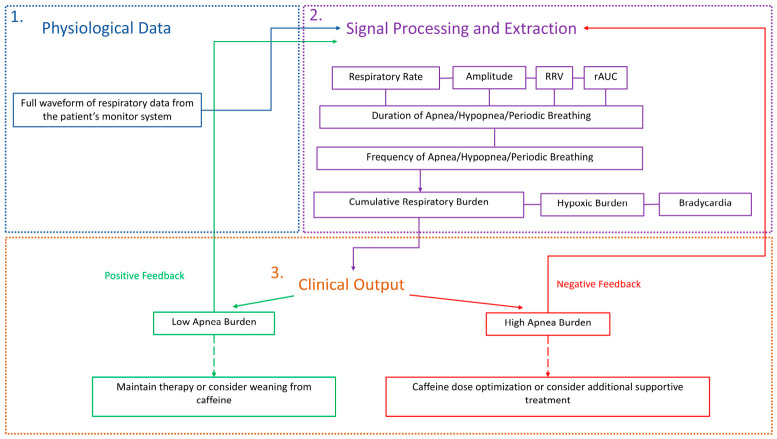
A proposed theoretical close loop of decision support algorithm using respiratory signals in caffeine treatment. RRV: respiratory rate variability, rAUC: area under the respiratory curve, CPAP: continuous positive airway pressure.

To support this paradigm shift, we propose a multi-layered theoretical framework designed to translate continuous respiratory monitoring into actionable clinical decision support (Figure 3). The framework consists of three interconnected stages: continuous data acquisition, transparent physiological signal processing, and clinical decision support. In the acquisition stage, multi-modal wearable biosensors continuously capture high-resolution physiological signals, including respiratory rate, rAUC, respiratory waveform amplitude and frequency changes, and the frequency and duration of apneic or hypopneic events. These signals may be integrated with complementary ECG and pulse oximetry measurements to provide a broader physiological context. The processing layer is designed to identify evolving respiratory trends and establish an adaptive physiological reference profile during periods of relative respiratory stability. However, because many neonates with AOP may not initially demonstrate a clearly stable individual baseline, future development of population-informed normative datasets may help contextualize physiological variability during the early stages of monitoring. By aggregating high-resolution respiratory data across large neonatal cohorts, such systems could support preliminary interpretation of clinical deterioration while progressively transitioning towards individualized baseline assessment over time.

Crucially, because the input metrics (e.g., rAUC) are derived from continuously accessible raw data, clinicians can visually verify the algorithm’s physiological rationale, ensuring the system remains transparent and continuously auditable. To maximize diagnostic specificity, this continuous analysis is dynamically cross-validated with heart rate and oxygen saturation monitoring. Requiring a precipitous drop in rAUC to temporally align with bradycardia or desaturation provides a highly robust, artifact-resistant indicator of AOP severity. By synthesizing these metrics, the system can identify pre-apnea events such as hypopnea or periodic breathing, shifting the monitoring paradigm from late-stage consequence detection to early physiological cause identification.

Finally, the decision support layer evaluates the cumulative respiratory and hypoxic burden to provide personalized clinical recommendations. For example, a clinically significant increase in respiratory burden may indicate inadequate caffeine plasma levels, prompting a potential dose escalation. Conversely, a stable profile supports maintaining the current dose or initiating weaning protocols. By calibrating continuously to the infant’s own physiological norm rather than static, population-based thresholds, this closed-loop strategy inherently mitigates the well-documented biases associated with ethnicity, skin tone, and gender observed in traditional monitors.

This approach aligns directly with the dual-biomarker framework proposed by Fatunla, et al. [2]. In their model, Category 1 biomarkers determine medication necessity, while Category 2 biomarkers optimize dosing and assess efficacy. Proxy monitoring addresses both by accurately capturing vital signs and preventing the underreporting of apneic events. This multimodal synthesis quantifies immediate pharmacodynamic responses and helps predict the risk of recurrent apnea, supporting precision medicine in neonatal care.

Consequently, these continuous physiological metrics may provide a more objective foundation for defining clinically meaningful thresholds for the initiation, titration, and discontinuation of caffeine therapy. Beyond individual patient management, standardization of these metrics could also support the development of large-scale international respiratory data repositories. By aggregating high-resolution physiological data across neonatal populations, such repositories may improve understanding of AOP pathophysiology and facilitate more robust evaluation of therapeutic strategies for caffeine treatment.

To bridge current clinical reality with the proposed framework, it is essential to compare their operational paradigms. The current consensus, as discussed in Section 2, is based on weight-adjusted dosing and the subjective evaluation of apnea severity through discrete apnea, bradycardia, and oxygen desaturation events. In contrast, this framework evaluates the clinical status of preterm infants using a broader set of continuous metrics including event durations, rAUC, RR, and hypoxic burden. Thus, this system can help to establish clearer start and end points for caffeine treatment, facilitate precise dosing adjustments, and enable the early detection of toxicity.

## 5. Limitation and Challenges

Despite the promising potential of this multi-modal framework, several challenges must be addressed for clinical implementation. The primary technical hurdle in the NICU environment is the prevalence of motion artifacts. Preterm infants frequently exhibit spontaneous movements, crying, and startle reflexes, which can introduce significant noise into kinematic sensor data, potentially leading to signal distortion. While our proposed integration of ECG and SpO_2_ serves as a cross-validation layer to mitigate false alarms, developing robust, motion-tolerant signal processing algorithms remain essential. Furthermore, the long-term skin integrity of wearable patches on the extremely fragile skin of premature neonates requires rigorous clinical validation.

Furthermore, profound anatomical and physiological differences exist between extremely preterm (<28 weeks) and very preterm (<32 weeks) infants. Specifically, lung development is typically in the canalicular phase (17–27 weeks), whereas the latter have advanced to the saccular phase (28–36 weeks) [89]. Consequently, applying a universal, static rAUC threshold across this wide developmental spectrum could lead to inaccurate clinical interpretations. Establishing gestational age-stratified or lung development-based normative datasets is therefore essential to ensure that continuous monitoring algorithms are appropriately calibrated to the specific anatomical and physiological maturity of each infant.

The practical implementation of a multi-modal system in the NICU presents significant system integration challenges. Continuous wearable biosensors are inherently susceptible to baseline drift over time, necessitating robust and dynamic calibration protocols. Simultaneous integration of multiple biosensors increases the risk of signal latency, synchronization errors, and intermittent data loss, all of which may affect the reliability of continuous real-time monitoring. Processing continuous physiological data also poses computational requirements. For this framework to be clinically viable and integrated into existing NICU infrastructure, a centralized, high-fidelity data integration platform is required to ensure temporal synchronization and continuous sensor calibration. Beyond these technical hurdles, clinician acceptance remains significant; therefore, AI driven metrics require transparent algorithms and clinical oversight.

In addition, deployment of continuous multi-sensor monitoring systems raises important regulatory, cybersecurity, and data privacy considerations, requiring compliance with international healthcare governance frameworks and medical device regulations.

Finally, prior to clinical adoption, the proposed algorithms and metrics must undergo rigorous external validation across large, multi-center neonatal cohorts to ensure safety, efficacy, and generalizability.

## 6. Conclusions and Future Suggestions

In conclusion, the clinical management of AOP must evolve from reactive, symptom-based interventions to proactive, data-driven optimization. While caffeine therapy remains the cornerstone of treatment, its full therapeutic potential is currently hindered by outdated, artifact-prone monitoring paradigms, and static dosing protocols. By integrating transparent wearable biosensors into neonatal care, proxy monitoring provides the objective, continuous pharmacodynamic biomarkers urgently needed to overcome the limitations of traditional black-box systems. This seamless integration of pharmacology and advanced sensor technology empowers clinicians to dynamically titrate caffeine doses based on an infant’s real-time respiratory response and cumulative burden, rather than relying solely on weight-based metrics.

To transition this conceptual framework into a practical bedside tool, future technological advancements must prioritize data transparency, enabling objective and auditable outcome analysis. Subsequent clinical research should focus on validating continuous proxy metrics, such as rAUC and respiratory rate variability, within the active NICU setting. This empirical validation should define evaluation criteria, especially validation, sensitivity, specificity, and receiver operating characteristic (ROC) analysis against to gold-standard monitoring systems in NICU. Crucially, these high-resolution respiratory biomarkers must be integrated with concomitant bradycardia and oxygen desaturation events to formulate a robust, multimodal diagnostic index. Furthermore, future iterations must incorporate advanced signal processing and artifact rejection mechanisms to distinguish true physiological variations from motion artifacts and environmental noise. Addressing these translational challenges necessitates a highly collaborative, multidisciplinary approach uniting clinical neonatologists, pharmacists, nurses, biomedical engineers, and data scientists. Ultimately, successfully validating these integrated systems will facilitate the critical shift from a conventional ‘one-size-fits-all’ approach to a true precision medicine framework, personalizing caffeine therapy to meet the unique physiological needs of every preterm infant.

## Figures and Tables

**Table 1:** Pharmacokinetic parameters in preterm infants following 20 mg/kg intravenous caffeine citrate loading dose followed by a maintenance of 5 mg/kg caffeine citrate once a day either by intravenous or oral administration.

Pharmacokinetic Parameter	Value
Absorption	Onset of action within minutes of commencement of infusion. Bioavailability after oral administration is 100% with time to peak plasma concentration 0.5–2 h. (slower after feeding). After an oral dose of 20 mg/kg caffeine citrate peak plasma concentration ranges from 6–10 mg/L.
Distribution	Mean volume of distribution is 0.8–0.9 L/kg. Rapid distribution into the brain following administration.
Metabolism	Limited metabolism in preterm infants due to limited expression of hepatic cytochrome P450 1A2. Most caffeine (~86%) is renally excreted as the parent compound. 3–8% of caffeine is converted to theophylline.
Elimination	Prolonged half-life of approximately 3–4 days
Plasma Level	8–40 mg/L

**Table 2:** Summary of the monitors for Apnea of Prematurity with strengths and limitations.

Monitor	Physiological Signal	Strengths	Limitations	References
ECG	Electrical heart activity	Accurate heart rate detection	Skin irritations from adhesives; restricted movements due to cables; delayed response; high false alarms	[45,46]
Pulse Oximetry	Photoplethysmogram (PPG)	Continuous estimation of peripheral oxygen saturation	Motion artifacts, skin pigmentation bias, sensor placement difference, weak correlation with arterial saturation	[47,50,53,54]
Thoracic Impedance	Chest wall movement (electrical resistance)	Widely used for detecting apneas and basic respiratory rate	Unable to detect obstructive sleep apnea, motion artifacts	[45,46,48]
Contactless Sensors	Motion/Micro-color changes	Eliminates skin irritations	Equipment cost, short duration validation, motion artifacts, presence direct skin	[59,60,61,62,63]

**Table 3:** Proxy metrics modal for apnea of prematurity monitoring. rAUC: area under the respiratory curve, ECG: electrocardiogram, RRV: respiratory rate variability, SpO_2_: peripheral oxygen saturation, AASM: American Academy of Sleep Medicine, AOP: Apnea of Prematurity, NICU: Neonatal Intensive Care Unit, PPG: photoplethysmography.

Physiological Metric	Sensor Modality	Signal Processing	Clinical Rational
Respiratory Rate	Kinematic Sensors (e.g., accelerometer, piezoelectric)	Peak-to-peak interval detection from continuous respiratory waveform and produce respiratory rate variability	Fundamental metric for detecting early clinical deterioration. While RR identifies precise pauses (based on missed respiratory cycles), RRV acts as a predictive biomarker; high variation serves as an early-warning indicator of neurological immaturity and impending AOP events before a gross apnea occurs.
Waveform Amplitude		Time-domain analysis of sub-baseline amplitudes drops to explicitly quantify the exact duration (seconds) and frequency (events/hour) of apneic/hypopneic episode.	AASM defines the complete apneic cessation (e.g., >90% drop) and clinically significant hypopneic episodes (e.g., >30% drop) in pediatrics. Similar criteria can apply for preterms to calculate respiratory burden.
rAUC		Continuous integration of respiratory waveform amplitude over time, calculated against a dynamic baseline.	Continuous, non-invasive proxy metric of breathing—analogous to a real-time spirometry assessment. By integrating the respiratory waveform over time, rAUC quantifies the total inspiratory effort and air exchange. A precipitous drop in rAUC provides a more sensitive early-warning indicator of impending apneic collapse than traditional respiratory rate monitoring alone, directly reflecting the decline in effective ventilation.
Bradycardia	Continuous Electrogram (ECG)	Automated detection of transient heart rate decelerations (R-R interval lengthening) concurrent with respiratory pauses.	Utilizes a standard, widely accepted NICU monitoring parameter. By integrating heart rate data with high-resolution kinematic respiratory metrics, the algorithm significantly reduces artifact-induced false alarms. This cross-validation ensures that alarms are only triggered when respiratory pauses lead to actual physiological compromise.
Hypoxic Burden	Photoplethysmography (PPG/pulse oximetry)	Integration of the area under the SpO_2_ curve below a patient-specific threshold (e.g., <3% drop).	Adopts an emerging metric from adult sleep studies to the preterm population. Unlike traditional desaturation counts, it quantifies the cumulative of hypoxia. This provides a more precise clinical correlation for neurodevelopmental risk and serves as a critical cross-validator for kinematic rAUC data.

## Data Availability

No new data were created or analyzed in this study.

## Abbreviations

The following abbreviations are used in this manuscript:

A	Amplitude
AAP	American Academy of Pediatrics
AASM	American Academy of Sleep Medicine
ANMF	Australasian Neonatal Medicines Formulary
AOP	Apnea of Prematurity
AUC	Area Under the Curve
BMP	Beats Per Minute
CAP	Caffeine Therapy for Apnea of Prematurity
CHIME	Caffeine for Hypoxic–Ischemic Encephalopathy
CPAP	Continuous Positive Airway Pressure
ECG	Electrocardiogram
FDA	Food and Drug Administration
IH	Intermittent Hypoxia
kg	Kilogram
L	Liter
mg	Milligram
NICE	National Institute for Health and Care Excellence
NICU	Neonatal Intensive Care Unit
PPG	Photoplethysmogram
rAUC	Area Under the Respiratory Curve
ROC	Receiver Operating Characteristic
RRV	Respiratory Rate Variability
SaO_2_	Arterial oxygen saturation
SpO_2_	Peripheral oxygen saturation
TDM	Therapeutic Drug Monitoring
WHO	World Health Organization
